# Single-cell and spatial profiling reveal an IL-10–associated iCAF–M2 macrophage communication axis in high-grade serous ovarian cancer ascites

**DOI:** 10.3389/fimmu.2026.1790912

**Published:** 2026-07-10

**Authors:** Ren Li, Jinquan Xia, Chang Zou, Yixia Xie, Yuan Shen

**Affiliations:** 1 The Second Clinical Medical College, Jinan University (Shenzhen People’s Hospital), Shenzhen, Guangdong, China; 2 Department of Clinical Research Centre, Shenzhen People’s Hospital (The First Affiliated Hospital, Southern University of Science and Technology; The Second Clinical Medical College, Jinan University), Shenzhen, Guangdong, China; 3 Department of Intensive Care Unit, Shenzhen People’s Hospital (The First Affiliated Hospital, Southern University of Science and Technology; The Second Clinical Medical College, Jinan University), Shenzhen, Guangdong, China; 4 Department of Gynecology, The First Affiliated Hospital of Jinan University, Guangzhou, Guangdong, China

**Keywords:** fibroblast–macrophage crosstalk, high-grade serous ovarian cancer, IL-10–IL-10RA signalling axis, immune-humanized PDX therapy, single-cell transcriptomics

## Abstract

**Aims and objectives:**

High-grade serous ovarian cancer (HGSOC) ascites represents a highly immunosuppressive tumour microenvironment characterized by complex stromal–immune interactions. In this study, we aimed to delineate the cellular communication networks shaping immune dysfunction within HGSOC ascites, identify key regulatory signalling pathways, characterize the fibroblast–macrophage axis at single-cell resolution, and evaluate the therapeutic potential of IL-10 blockade in reversing macrophage-mediated immunosuppression.

**Methods:**

Single-cell RNA sequencing data of eight ascites samples from six HGSOC patients (GSE146026) were analysed using Seurat for clustering and differential expression, followed by CellChat to infer ligand–receptor communication. Sub-clustering defined inflammatory CAFs (iCAF), myofibroblast CAFs (mCAF), and macrophage subtypes (M1/M2). GO/KEGG enrichment assessed functional pathways. Multiplex immunofluorescence validated spatial interactions of IL10+ iCAF and IL10RA+ macrophages in patient tissues. An immune-humanized patient-derived xenograft (PDX) mouse model was established to evaluate the therapeutic effect of IL-10 blockade.

**Results:**

Single-cell transcriptomic analysis of HGSOC ascites revealed macrophages and cancer-associated fibroblasts as dominant cell populations within the tumour microenvironment. Cell–cell communication analysis identified IL-10–IL-10RA signalling as a prominent interaction pathway linking inflammatory CAFs (iCAFs) with M2-like macrophages. iCAFs displayed elevated IL-10 expression, while M2 macrophages showed increased IL-10RA/IL-10RB expression and were associated with exhausted immune states. Multiplex immunofluorescence confirmed spatial proximity between IL-10^+^ iCAFs and IL-10RA^+^ M2 macrophages in tumour tissues. In immune-humanized PDX models, IL-10 blockade with MK-1966 significantly suppressed tumour growth and reduced expression of M2 macrophage markers and proliferation-associated proteins.

**Conclusion:**

This study identifies an IL-10-dependent iCAF–M2 macrophage immunoregulatory circuit as a central mechanism driving immune suppression in HGSOC. Targeting the IL-10 pathway effectively reverses M2 polarization and suppresses tumour progression, highlighting IL-10 blockade as a promising therapeutic strategy in ovarian cancer.

## Introduction

Ovarian cancer is a major global health burden, ranking among the leading causes of gynaecologic cancer mortality worldwide. Recent GLOBOCAN estimates reported approximately 324,000 new cases and 207,000 deaths annually, reflecting its disproportionately high mortality relative to incidence due to late-stage diagnosis and limited early detection strategies (1, 2). Epithelial ovarian cancer constitutes nearly 90% of all ovarian malignancies, with high-grade serous ovarian cancer (HGSOC) representing the predominant histological subtype, accounting for ~70% of cases and the majority of deaths (3, 4). HGSOC is frequently diagnosed at advanced FIGO stage III–IV in more than 70% of patients, resulting in poor clinical outcomes and persistently low 5-year survival rates despite therapeutic advances (5). Malignant ascites is the pathological accumulation of fluid within the peritoneal cavity and is a common clinical manifestation of advanced-stage HGSOC. Ascitic fluid represents a dynamic tumour-associated microenvironment that contains tumour cells, immune cells, stromal cells, soluble cytokines, and extracellular vesicles. Within this compartment, immune cells including macrophages, T lymphocytes, dendritic cells, and myeloid-derived cells interact with tumour and stromal elements to regulate tumour progression, immune escape, and therapeutic resistance. Among these populations, tumour-associated macrophages (TAMs) and cancer-associated fibroblasts (CAFs) are particularly abundant and play central roles in shaping the immunosuppressive microenvironment through cytokine secretion, extracellular matrix remodelling, and paracrine signalling (5, 6). Single-cell RNA sequencing (scRNA-seq) has transformed our understanding of tumor heterogeneity and cellular ecosystems in HGSOC. Multiple studies using scRNA-seq have revealed complex ecosystems of tumor, stromal, and immune cells in HGSOC, identifying distinct cell subpopulations and ligand-receptor interactions that contribute to disease progression and therapeutic resistance (7, 8). In malignant ascites, the macrophage compartment is heterogeneous and may arise from both circulating monocytes recruited from peripheral blood and tissue-resident macrophages that migrate into the peritoneal cavity. These recruited monocytes can differentiate into tumour-associated macrophages under the influence of tumour-derived cytokines and stromal signals within the ascitic microenvironment. Such macrophages frequently acquire an M2-like immunosuppressive phenotype, contributing to immune evasion and tumour progression (9). TAMs often acquire an immunosuppressive M2-like phenotype characterized by high expression of CD163 and CD206, elevated production of IL-10, TGF-β, CCL18, and VEGF, and suppression of cytotoxic T-cell functions; these features correlate with poor outcomes in ovarian cancer patients (10). Concurrently, CAFs represent a major stromal cell population and exhibit functional heterogeneity, including iCAFs that secrete IL-6, CXCL12, and IL-8, and mCAFs enriched for ACTA2, TAGLN, and RGS5 expression, both of which contribute to extracellular matrix remodelling, immune suppression, and tumor progression (11, 12). IL-10 is a key immunoregulatory cytokine that exerts potent anti-inflammatory effects by signalling through its heterodimeric receptor composed of IL10RA and IL10RB. IL-10 signalling activates JAK1 and TYK2, leading to STAT3 phosphorylation and downstream transcriptional programs that suppress antigen presentation and effector T-cell activity (13). Several studies have demonstrated that IL-10 is markedly elevated in ovarian cancer ascitic fluid compared with serum and benign fluids, and higher ascitic IL-10 levels are significantly associated with decreased progression-free and overall survival in patients with advanced epithelial ovarian cancer (14). Myeloid cells including monocytes, macrophages, and dendritic cells are the predominant producers of IL-10 in ovarian tumor ascites, providing an immunosuppressive milieu that inhibits autologous T-cell proliferation and cytokine expression (15). These observations position IL-10 as a critical mediator of immune tolerance in the ovarian cancer microenvironment and a compelling candidate for therapeutic targeting.

Despite the recognized role of IL-10 in ovarian cancer, the cellular sources, receptor-expressing target cells, and specific intercellular communication pathways underpinning IL-10-driven immune suppression have not been thoroughly elucidated at single-cell resolution. Here, we leveraged single-cell transcriptomics and cell–cell communication analysis to characterize the IL-10–cantered immunoregulatory network within HGSOC ascites. Our study identifies inflammatory CAFs (iCAFs) and M2-like macrophages as key signalling partners within this pathway and evaluates the therapeutic potential of IL-10 blockade using an immune-humanized patient-derived xenograft model.

## Methods

### Ethical approval and human samples

All experimental procedures involving human participants and clinical samples were conducted in accordance with the ethical standards of the institutional and national research committees and followed the principles outlined in the Declaration of Helsinki (revised 2013). Ovarian cancer tissue samples were obtained from patients with pathologically confirmed HGSOC who underwent surgical resection at Shenzhen People’s Hospital. Written informed consent was obtained from all patients prior to sample collection. The study protocol was reviewed and approved by the Institutional Ethics Committee of Shenzhen People’s Hospital (Approval No.: LL-KY-2024107-04). All animal experiments were performed in accordance with the Guide for the Care and Use of Laboratory Animals and followed the ethical guidelines established by the National Institutes of Health. Experimental protocols were approved by the Institutional Animal Care and Use Committee (IACUC) of Shenzhen People’s Hospital (Approval No.: AUP-250515-ZC-0474-01). Animals were maintained under specific pathogen-free (SPF) conditions with controlled temperature and humidity under a 12-hour light/dark cycle with free access to food and water. Humane endpoints were strictly followed to minimize animal suffering.

### Data acquisition, quality control, and cell type identification

scRNA-seq data were obtained from 8 ascitic fluid specimens collected from 6 patients diagnosed with HGSOC. These samples were publicly available in the Gene Expression Omnibus (GEO) database https://www.ncbi.nlm.nih.gov/geo/ (Accession: GSE146026). The dataset comprises ascites isolated at primary diagnosis prior to chemotherapy, representing heterogeneous tumor microenvironment (TME) cellular states across patients. As the sequencing data were generated in the original study, the experimental procedures for ascites processing, cell isolation, and library preparation followed the protocols described in the original publication associated with the dataset. Raw 10x Genomics files (FASTQ and cell-barcoded UMI matrices) were processed using the Seurat (v3.2.1) package in R (v3.6.3). Initial quality control excluded cells with fewer than 200 detected genes (to remove low complexity/empty droplets), cells with more than 6,000 detected genes (to avoid doublets or multiplets), and cells with >10% mitochondrial gene expression (indicative of poor cell viability). Technical doublets were further filtered using DoubletFinder with default parameters (16), ensuring removal of spurious clusters caused by multiple cells erroneously captured in a single droplet. After QC, UMI counts were normalized using the “LogNormalize” method in Seurat, scaling each cell to 10,000 transcripts and log-transforming the data to stabilize variance. To account for batch effects across patients and sequencing runs, samples were integrated using Seurat’s canonical correlation analysis (CCA) and Find Integration Anchors/Integrate Data workflow. Highly variable genes were identified using the “vst” selection method, and these were used for downstream dimensionality reduction.

Principal component analysis (PCA) was performed on the integrated dataset, and significant principal components were selected based on ElbowPlot, JackStraw, and inspection of variance explained (>80% cumulative variance). Clustering was conducted using the Louvain algorithm with a resolution parameter optimized through cluster stability metrics. Dimensionality reduction for visualization was performed using Uniform Manifold Approximation and Projection (UMAP), which provides improved separation of biologically distinct clusters compared with tSNE. Cell clusters were annotated based on canonical marker gene expression (e.g., PTPRC for immune cells, CD68/CD163 for macrophages, PDGFRA/ACTA2 for fibroblasts, EPCAM for tumor cells, CD3D/CD3E for T-cells, MS4A1 for B cells, CEACAM8 for neutrophils), in combination with previously published ovarian cancer single-cell atlases (6, 16). Differential gene expression between clusters was determined using Seurat’s Find All Markers with thresholds: log_FC_ ≥ 0.25, min.pct ≥ 0.25, and min.diff.pct ≥ 0.25.

### Cell–cell communication analysis

Cell–cell communication networks were inferred using the CellChat R package (v1.4.0), which predicts biologically meaningful ligand–receptor–mediated signalling events based on curated human interaction databases (17). The normalized and batch-corrected Seurat object was converted into a CellChat object, and the human CellChatDB database was used as the ligand–receptor reference. Lowly expressed ligands and receptors (expression detected in<10% of cells in a cluster) were filtered to avoid spurious interactions. CellChat employs a probabilistic inference framework and permutation testing to quantify interaction probability and communication strength between cell populations. Global communication networks were constructed, and both outgoing signalling strength (sender activity) and incoming signalling strength (receiver activity) were computed for each cell type. The signalling pathways were aggregated to identify dominant intercellular communication axes. Only ligand–receptor interactions and signalling pathways with *p<* 0.05 (permutation-corrected) were retained as statistically significant.

### Functional enrichment analysis

DEGs were subjected to functional enrichment using clusterProfiler (v3.18.1) (18). Gene Ontology (GO; Biological Process, Cellular Component, and Molecular Function) and Kyoto Encyclopaedia of Genes and Genomes (KEGG) pathway analyses were performed using org.Hs.eg.db as the annotation reference. Enrichment was performed using over-representation analysis (ORA), with the background gene list defined as all expressed genes in the dataset to avoid sampling bias. Significantly enriched biological categories were identified using the statistical thresholds: *p*-value< 0.05; Benjamini–Hochberg (BH) adjusted q-value< 0.05; Minimum gene set size ≥10 genes. Pathways failing multiple-testing correction were excluded. Enrichment results were visualized using dot plots and enrichment maps to highlight pathway hierarchy and functional clustering. For FDR in GSCA immune infiltration analysis, the original P-value was calculated and sorted from small to large, and then calculate the corrected Q-value (FDR) based on the ranking of each P-value and the total number of tests using the formula.


FDR=(P×N)/rank.

### Multiplex immunofluorescence staining

Tumour tissue samples were obtained from patients with pathologically confirmed high-grade serous ovarian cancer who underwent surgical resection at Shenzhen People’s Hospital. Tumour specimens were collected from the primary ovarian tumor site during cytoreductive surgery prior to systemic therapy. Tumour tissues were fixed in 4% paraformaldehyde for ≤48 h at 4 °C, dehydrated, paraffin-embedded, and sectioned at 4 μm thickness. Sections were deparaffinized, rehydrated, and subjected to heat-induced antigen retrieval using citrate buffer (pH 6.0) in a pressure chamber. Endogenous peroxidase activity was quenched and non-specific binding was blocked according to manufacturer instructions. Multiplex staining was performed using the PANO 5-plex IHC kit (Panovue, Beijing, China) combined with Bond Polymer Refine Detection (DS9800-CN) and Bond Polymer Refine Red Detection (DS9390) on a BOND RX automated platform (Leica Biosystems) to ensure standardized staining conditions. Fluorophore channels were assigned to avoid spectral overlap. Primary antibodies included: IL-10 (Abclonal, A2171; 1:100); IL-10RA (Proteintech, 13356-1-AP; 1:200); PDGFRA (Proteintech, 82943-1-RR; 1:100); CD206 (Proteintech, 18704-1-AP; 1:200). Slides were counterstained with DAPI and scanned using Olympus VS200 at identical exposure parameters. Negative controls (isotype and secondary-only) were included in all runs to exclude nonspecific staining. Quantitative analysis was performed using inform image analysis software (Version 2.4, PerkinElmer, Waltham, Massachusetts, US). Five representative regions of interest (ROIs) measuring 1 mm² each were selected per tumour section. All ROIs were manually annotated by two independent, investigators who were blinded to all clinical, outcome, and single-cell sequencing data. To assess the reproducibility of the manual annotations, the overlap or correlation between the two investigators’ annotations was evaluated. Any discrepancies (e.g., differences in area demarcation or inclusion/exclusion of ambiguous regions) were resolved by a consensus review between the two investigators and a third senior pathologist if needed. In response to the blind peer reviewers' request to supplement experimental data, the following describes the experimental methods for the PDX model. Immunofluorescence staining of mouse tissues was performed as described previously. The primary antibodies used were: Ki67 (Abcam, ab16667; 1:200) and EpCAM (Abcam, ab71916; 1:200).

### Immune-humanized PDX mouse model and IL-10 blockade treatment

Female NSG Rag2−/− IL2rg−/− mice (4–6 weeks old) were obtained from GemPharmatech (Nanjing, China) and maintained under specific pathogen-free conditions. Human peripheral blood mononuclear cells (PBMCs) (1*10^7^ cells per mouse) from 12 healthy donors (6 male, 6 female) were isolated and co-administrated with THP-1 cells induced to become macrophages (3*10^4^ cells per mouse) to generate immune-humanized mice. Patient-derived ovarian cancer tissues were obtained from primary ovarian lesion, dissected in a sterile petri dish to remove necrotic areas The humanized mice were anesthetized via isoflurane inhalation. The right flank was shaved and sterilized with povidone-iodine. A small (~5 mm) longitudinal incision was made in the skin of the right flank. A subcutaneous pocket was created by blunt dissection using sterile forceps. A single tumor fragment was then placed into this pocket. The incision was closed with a 5–0 absorbable suture and sealed with tissue glue. Once tumors reached ~100 mm³, animals were randomized into: Control group (vehicle) and MK-1966 treatment group (anti-IL-10 monoclonal antibody; MCE, HY-P991257). MK-1966 was administered at 2 mg/kg, intraperitoneal, every 3 days for the treatment period. Randomization and treatment allocation were blinded. humane endpoints included excessive weight loss (>20%), ulceration, impaired mobility, or tumor burden exceeding ethical limits. Tumor growth was monitored every 3 days and calculated as: $V=\frac{L\times W^{2}}{2}$ where L = longest diameter and W = shortest diameter. At study endpoint, tumors were excised, weighed, photographed, and harvested for immunohistochemistry. Animals that died or met humane-endpoint criteria prior to study completion were excluded from final growth analysis but retained for survival analysis. Group size was determined based on previous experience in similar xenograft models to ensure adequate power.

### Statistical analysis

Statistical analyses were performed using GraphPad Prism 10.0. Data are expressed as mean ± standard deviation (SD). Tumour growth curves were analysed using two-way repeated-measures ANOVA with *post-hoc* correction where appropriate. Comparisons between two groups were assessed using unpaired Student’s t-test following normality assessment. A *p* < 0.05 was considered statistically significant. All analyses were performed in a blinded manner.

## Results

### Single-cell landscape of the HGSOC ascitic tumor microenvironment

**Figure 1:**
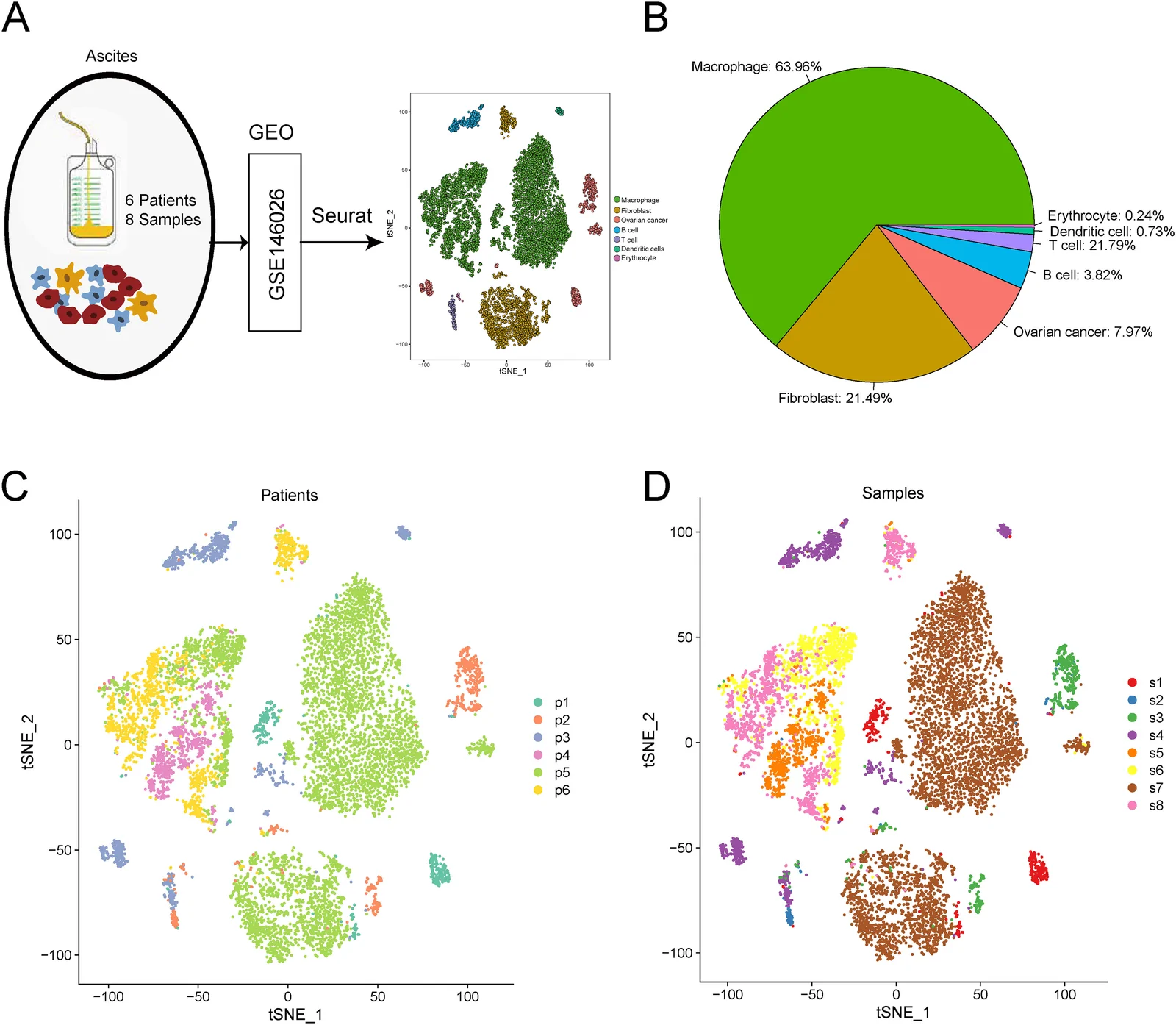
Single-cell landscape of the HGSOC ascitic tumor microenvironment. **(A)** Illustration showing the data from GEO (GSE146026) with 8 ascitic fluid specimens collected from 6 patients, including macrophages, cancer-associated fibroblasts (CAFs), ovarian cancer epithelial cells, T cells, B cells, dendritic cells, and erythrocytes. **(B)** Pie plot illustrating the composition across identified cell types. **(C)** t-SNE plot showing cells from the six patients, demonstrating inter-patient heterogeneity. **(D)** t-SNE plot showing the eight ascites samples, highlighting sample-level variability in immune and stromal composition.

Single-cell transcriptional analysis of 9,428 cells from eight malignant ascites samples obtained from six patients with HGSOC resolved the cellular composition of the ascitic tumour microenvironment into seven discrete clusters, including macrophages, cancer- CAFs, ovarian cancer epithelial cells, T cells, B cells, dendritic cells, and erythrocytes (**Figure 1A**). Clinical information, including patient demographics and sample collection details, are available in the original study describing the dataset (6). Macrophages represented the largest cellular compartment, followed by CAFs as the dominant stromal population, while tumor epithelial cells constituted another major proportion of the cellular pool (**Figure 1B**). In contrast, adaptive immune populations (T-cells and B-cells), dendritic cells, and erythrocytes comprised smaller fractions of the cellular landscape. When examined at the individual patient level, the contribution of these cell populations differed markedly across the six patients, with variable representation of macrophages, CAFs, and tumour epithelial cells, indicating pronounced inter-patient heterogeneity (**Figure 1C**). Evaluation across the eight individual ascites samples similarly demonstrated sample-dependent variation in cellular composition, with fluctuations in immune and stromal cell abundance between samples from different patients as well as between samples derived from the same patient (**Figure 1D**). Observed variability in cell-type abundance between samples reflects biological heterogeneity of the ascitic tumour microenvironment rather than analytical bias.

### IL10–IL10RA signalling defines a dominant fibroblast–macrophage communication axis

**Figure 2:**
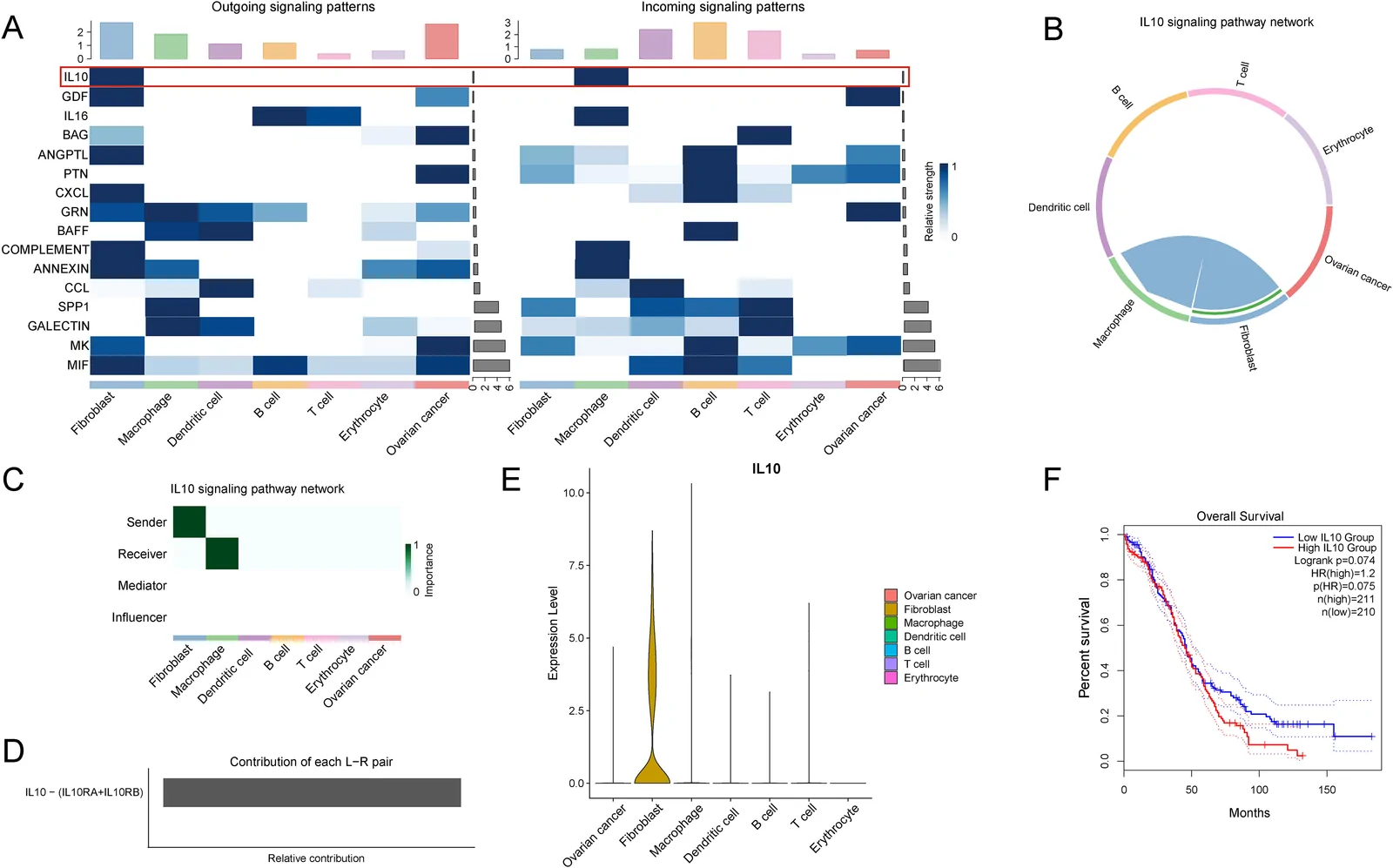
IL10–IL10RA signalling defines a dominant fibroblast–macrophage communication axis. **(A)** Heatmap depicting outgoing and incoming signalling strengths across cell populations, identifying IL-10 signalling as selectively enriched between fibroblasts and macrophages. **(B)** Circle network plot illustrating IL10 signalling connections, showing preferential communication between fibroblasts and macrophages. **(C)** Role analysis demonstrating fibroblasts as primary IL10 signal senders and macrophages as major signal receivers. **(D)** Contribution analysis confirming IL10–IL10RA/IL10RB as the dominant ligand–receptor pair mediating this pathway. **(E)** Violin plot showing elevated IL10 expression predominantly in fibroblasts. **(F)** Kaplan–Meier survival analysis from TCGA ovarian cancer cohort indicating poorer overall survival tendency in patients with higher IL10 expression.

Cell–cell communication analysis demonstrated that IL-10 signalling emerged as a distinct and highly enriched pathway within the tumour microenvironment, showing a strong directional interaction between fibroblasts and macrophages (**Figure 2A**). Among multiple signalling programs evaluated, IL-10 signalling displayed selectively elevated outgoing strength in fibroblasts and preferential incoming responsiveness in macrophages, indicating a uniquely focused signalling axis between these two populations (**Figures 2A, B**). Network role analysis further revealed that fibroblasts functioned predominantly as the principal IL10 signal senders, whereas macrophages represented the major IL10 signal receivers within the pathway architecture (**Figure 2C**). Contribution analysis confirmed that this interaction was primarily mediated by the IL-10–IL-10RA/IL-10RB ligand–receptor pair, accounting for the dominant strength of the pathway (**Figure 2D**). Network role analysis indicated that fibroblasts acted as the principal IL-10 signal senders, whereas macrophages served as the major signal receivers, consistent with macrophage expression of the IL-10 receptor complex (IL10RA/IL10RB). Consistent with this, expression profiling demonstrated that IL-10 was highly expressed in fibroblasts, whereas macrophages exhibited higher receptor expression signatures, supporting a fibroblast-to-macrophage signalling directionality (**Figure 2E**). Survival evaluation using the TCGA ovarian cancer cohort showed that patients with high IL-10 expression exhibited a trend toward poorer overall survival, whereas those with lower IL-10 expression demonstrated relatively improved survival outcomes, although not reaching strict statistical significance at the threshold displayed (**Figure 2F**). Present results define IL-10–IL-10RA signalling as a focused and dominant communication pathway between fibroblasts and macrophages within the HGSOC tumor microenvironment.

### Fibroblast IL10 expression aligns with IL10RA/IL10RB-enriched M2 macrophages

**Figure 3:**
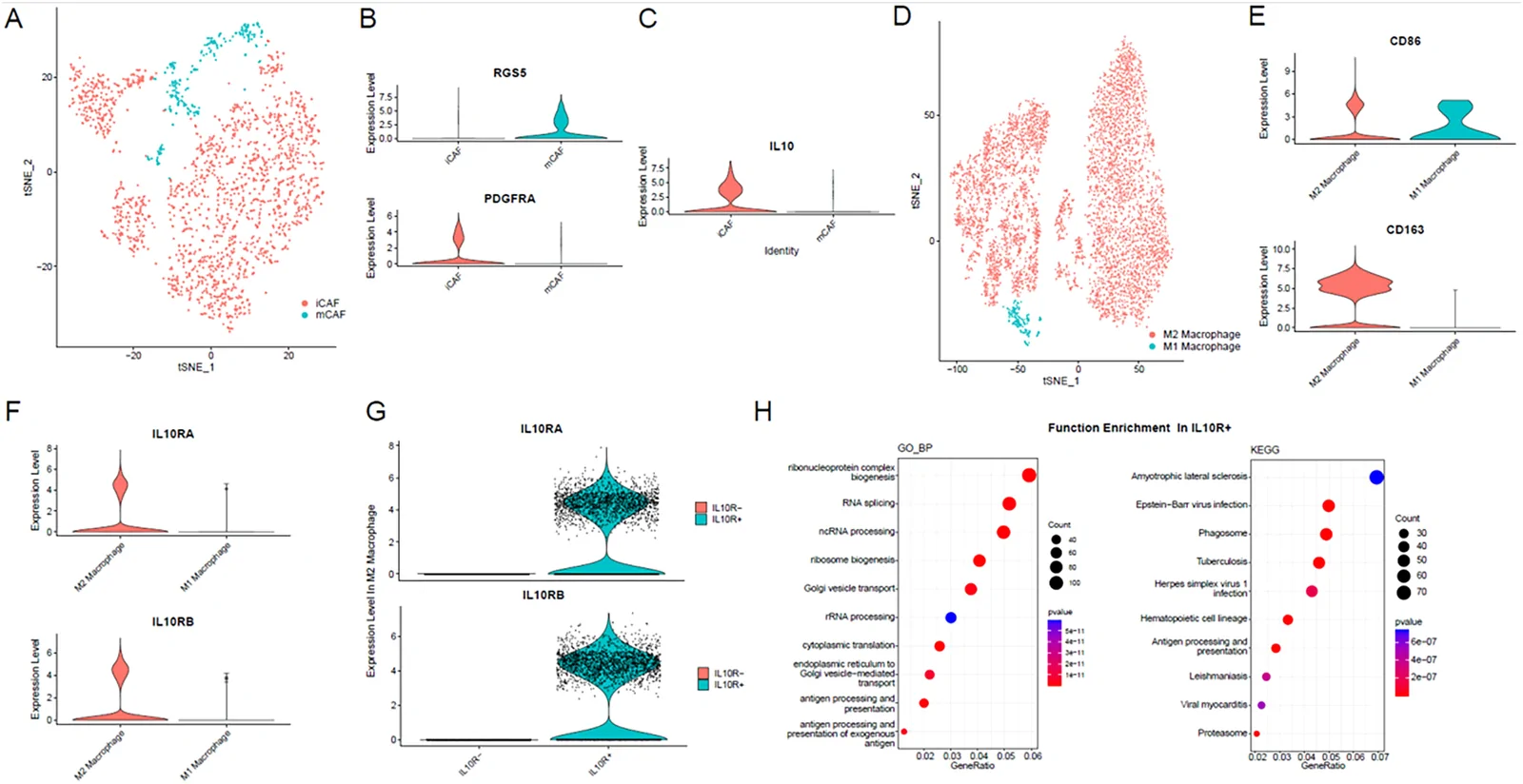
IL10-producing iCAFs align with IL10RA/IL10RB-enriched M2 macrophages. **(A)** t-SNE visualization showing the separation of fibroblast populations into inflammatory CAFs (iCAFs) and myofibroblastic CAFs (mCAFs). **(B)** Violin plots showing marker gene expression used to define fibroblast subtypes, with RGS5 enriched in mCAFs and PDGFRA enriched in iCAFs. **(C)** Violin plot showing IL10 expression predominantly in iCAFs, whereas mCAFs exhibit minimal expression. **(D)** t-SNE plot demonstrating segregation of macrophages into M1- and M2-like subsets. **(E)** Violin plots showing CD86 enrichment in M1 macrophages and CD163 enrichment in M2 macrophages, confirming macrophage polarization states. **(F)** Violin plots showing IL10RA and IL10RB expression elevated in M2 macrophages compared with M1 macrophages. **(G)** Stratification of macrophages based on IL10 receptor expression identifying an IL10R-high M2 macrophage subpopulation. **(H)** Gene Ontology (GO) biological process and KEGG pathway enrichment analyses of genes upregulated in IL10R-high M2 macrophages, highlighting enrichment of pathways related to antigen processing and presentation, ribonucleoprotein complex biogenesis, Golgi transport, and phagosome-associated immune processes. In violin plots, gene expression levels are represented by the width of the distribution, where wider regions indicate a higher density of cells expressing the gene at that level.

t-SNE analysis of fibroblast populations resolved two distinct subsets corresponding to iCAFs and mCAFs (**Figure 3A**). Violin plot analysis confirmed RGS5 enrichment in mCAFs and PDGFRA enrichment in iCAFs, validating subtype identity (**Figure 3B**). Among these subpopulations, IL-10 expression was predominantly elevated in iCAFs, whereas mCAFs exhibited minimal IL-10 expression (**Figure 3C**). Macrophages were likewise segregated into two major subsets, consistent with M1 and M2 phenotypic states (**Figure 3D**). CD86 expression characterized M1 macrophages, whereas CD163 expression was enriched in M2 macrophages (**Figure 3E**). Within these macrophage subsets, IL10RA and IL10RB expression was selectively elevated in M2 macrophages, with minimal expression observed in M1 macrophages (**Figure 3F**). Further stratification demonstrated that macrophages with high IL10RA/IL10RB expression formed a distinct IL10R-positive subgroup within the M2 compartment (**Figure 3G**). Functional enrichment analysis of genes upregulated in IL10R-positive M2 macrophages revealed enrichment of pathways associated with antigen processing and presentation, ribonucleoprotein complex biogenesis, Golgi transport processes, and multiple immune-associated KEGG pathways including phagosome, antigen presentation pathways, viral infection pathways, and proteasome activity (**Figure 3H**). Findings demonstrate that IL-10 is predominantly produced by iCAFs, while M2-macrophages preferentially express IL-10RA/IL-10RB, supporting a selective alignment between IL-10-producing fibroblasts and IL-10-responsive macrophage subsets within the HGSOC microenvironment. Although pathways related to antigen processing and phagosome activity were enriched, these functions can also occur in tumour-associated macrophages with immunoregulatory phenotypes and do not necessarily indicate classical pro-inflammatory activation.

### IL-10R-high M2 macrophage signature associates with exhausted immunity and macrophage enrichment

**Figure 4:**
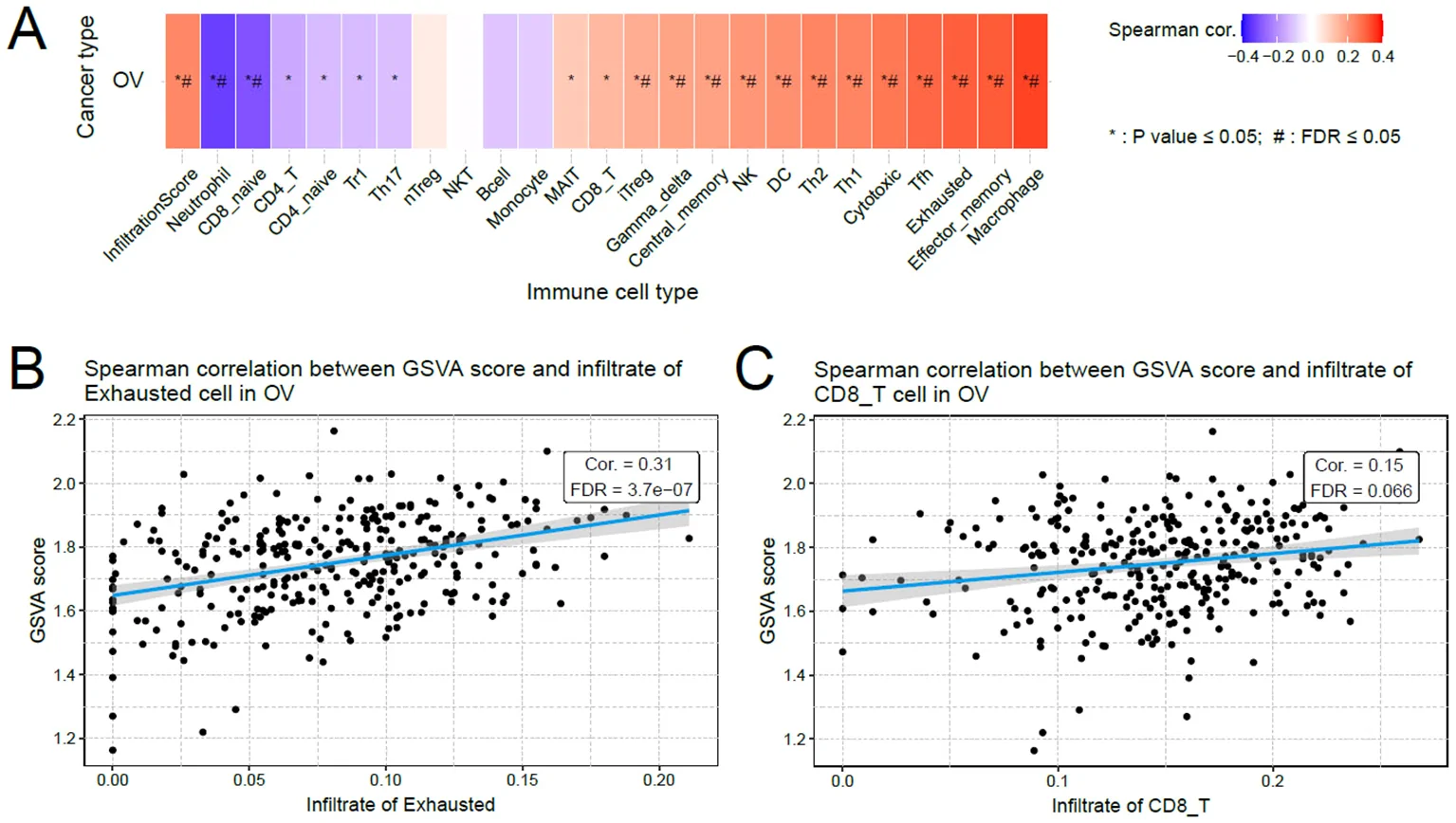
IL10R-high M2 macrophage signature correlates with exhausted and macrophage-enriched immune states. **(A)** Correlation heatmap showing strong positive associations between the IL10RA/IL10RB-high M2 macrophage signature and exhausted T cells, effector memory T cells, and macrophages. **(B)** Scatter plot showing a significant positive correlation between the gene signature and exhausted T-cell infiltration (Spearman r = 0.31; FDR = 3.7×10^-7^). **(C)** Scatter plot showing a weaker and nonsignificant correlation between the gene signature and CD8^+^ T-cell infiltration (Spearman r = 0.15; FDR = 0.066).

Correlation analysis between the IL-10RA/IL-10RB-high M2-macrophage gene signature and immune infiltration patterns in ovarian cancer demonstrated selective and strong positive associations with immunoregulatory and suppressive immune states (**Figure 4A**). Across immune cell categories, the signature showed the highest positive correlations with exhausted T cells, effector memory T cells, and macrophages, whereas correlations with naïve T-cell subsets were weaker. A significant positive correlation was observed between the IL10R-high M2 signature and exhausted T-cell infiltration (Spearman r = 0.31; FDR = 3.7×10^-7^), indicating a close association between this macrophage phenotype and T-cell exhaustion levels in ovarian cancer (**Figure 4B**). In comparison, the correlation with CD8^+^ T-cell infiltration was weaker (Spearman r = 0.15) and did not meet the false discovery rate significance threshold (FDR = 0.066), indicating that the association of the signature is more strongly aligned with T-cell exhaustion than with total CD8^+^ T-cell presence (**Figure 4C**). Together, these results demonstrate that the IL10RA/IL10RB-high M2 macrophage transcriptional program is preferentially associated with exhausted immune states and macrophage-rich microenvironments, rather than with cytotoxic CD8^+^ T-cell infiltration.

### IL10^+^ iCAFs spatially engage IL10RA^+^ M2 macrophages within tumor tissue

**Figure 5:**
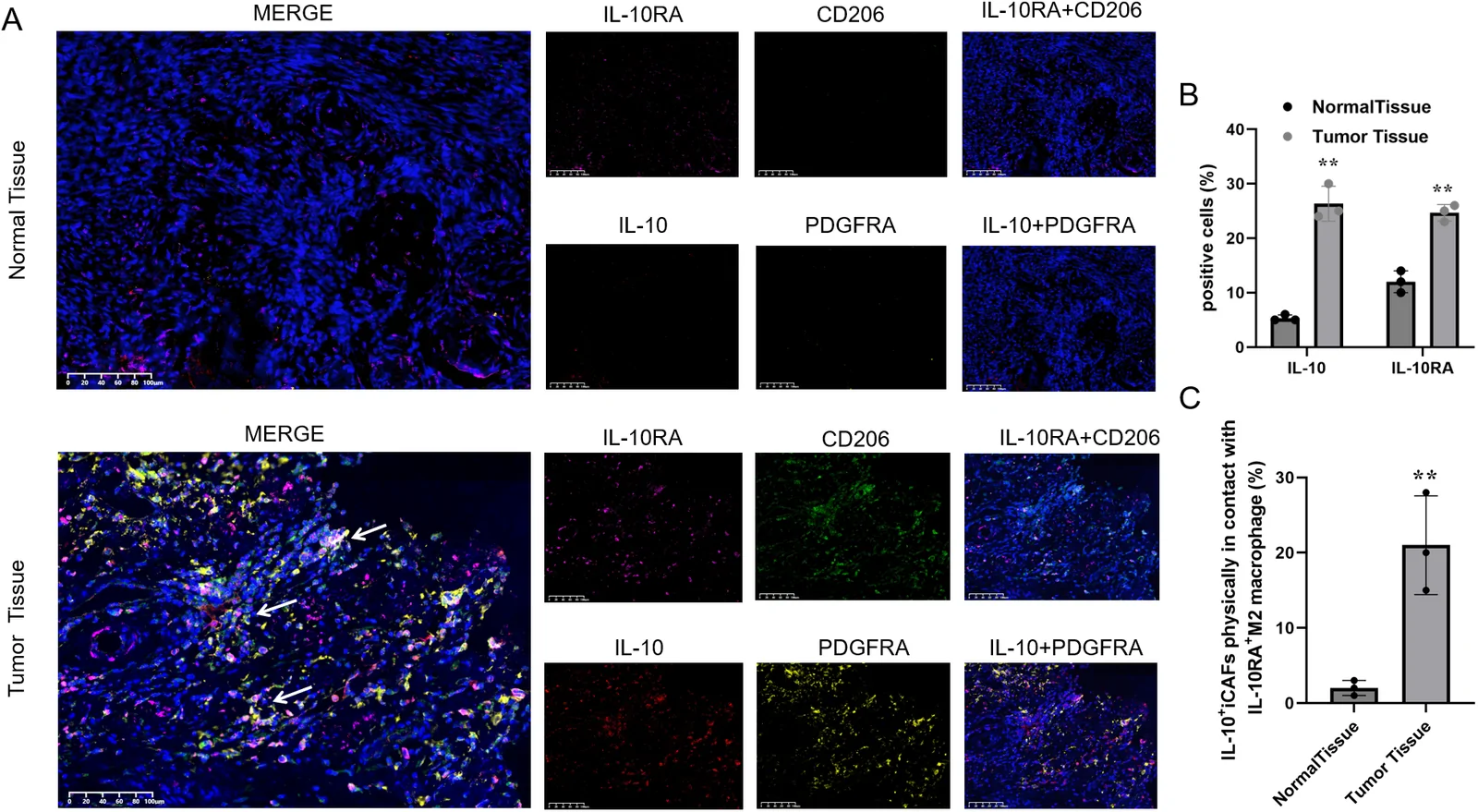
Spatial colocalization of IL-10^+^ iCAFs and IL-10RA^+^ M2 macrophages in tumor tissue. **(A)** Multiplex immunofluorescence images showing minimal IL-10, IL-10RA, CD206, and PDGFRA staining in normal tissue (upper panel), contrasted with marked IL10^+^ fibroblast and IL10RA^+^CD206^+^ macrophage abundance and spatial adjacency in tumor tissue (lower panel; arrows). **(B)** Quantitative comparison showing significantly higher IL-10^+^ and IL-10RA^+^ cell proportions in tumor tissue compared with normal tissue (*p* < 0.01). **(C)** Increased frequency of physical contact between IL-10^+^ iCAFs and IL-10RA^+^ M2 macrophages in tumor tissue relative to normal tissue (*p* < 0.01). ** represent p-value < 0.01.

Multiplex immunofluorescence analysis demonstrated minimal IL10 and IL10RA staining in normal ovarian tissue, with very low detection of CD206^+^ macrophages and PDGFRA^+^ fibroblasts (**Figure 5A**, upper panel). In contrast, tumor tissue exhibited markedly increased IL-10 expression and pronounced IL10RA positivity, together with abundant CD206^+^ macrophages and PDGFRA^+^ fibroblasts (**Figure 5A**, lower panel). Co-localization panels revealed frequent IL-10RA^+^CD206^+^ macrophages and IL10^+^PDGFRA^+^ fibroblasts within tumor sections. Quantitative analysis confirmed a significantly higher percentage of IL-10^+^ and IL-10RA^+^ cells in tumor tissue compared with normal tissue (both *p* < 0.01; **Figure 5B**). Spatial interaction assessment further demonstrated that IL10^+^ iCAFs were in close physical contact with IL10RA^+^ M2 macrophages at a substantially higher frequency in tumor regions than in normal tissue (*p* < 0.01; **Figure 5C**). Representative fields highlighted regions of dense spatial juxtaposition between IL-10-producing fibroblasts and IL10RA-expressing macrophages within tumor stroma (**Figures 5A**, arrows). These observations show that tumor tissue contains increased IL-10^+^ iCAFs, elevated IL-10RA^+^ M2 macrophages, and significantly enhanced spatial proximity between these two cell populations, compared with normal ovarian tissue.

### IL10 blockade with MK-1966 suppresses tumor growth in humanized PDX models

**Figure 6:**
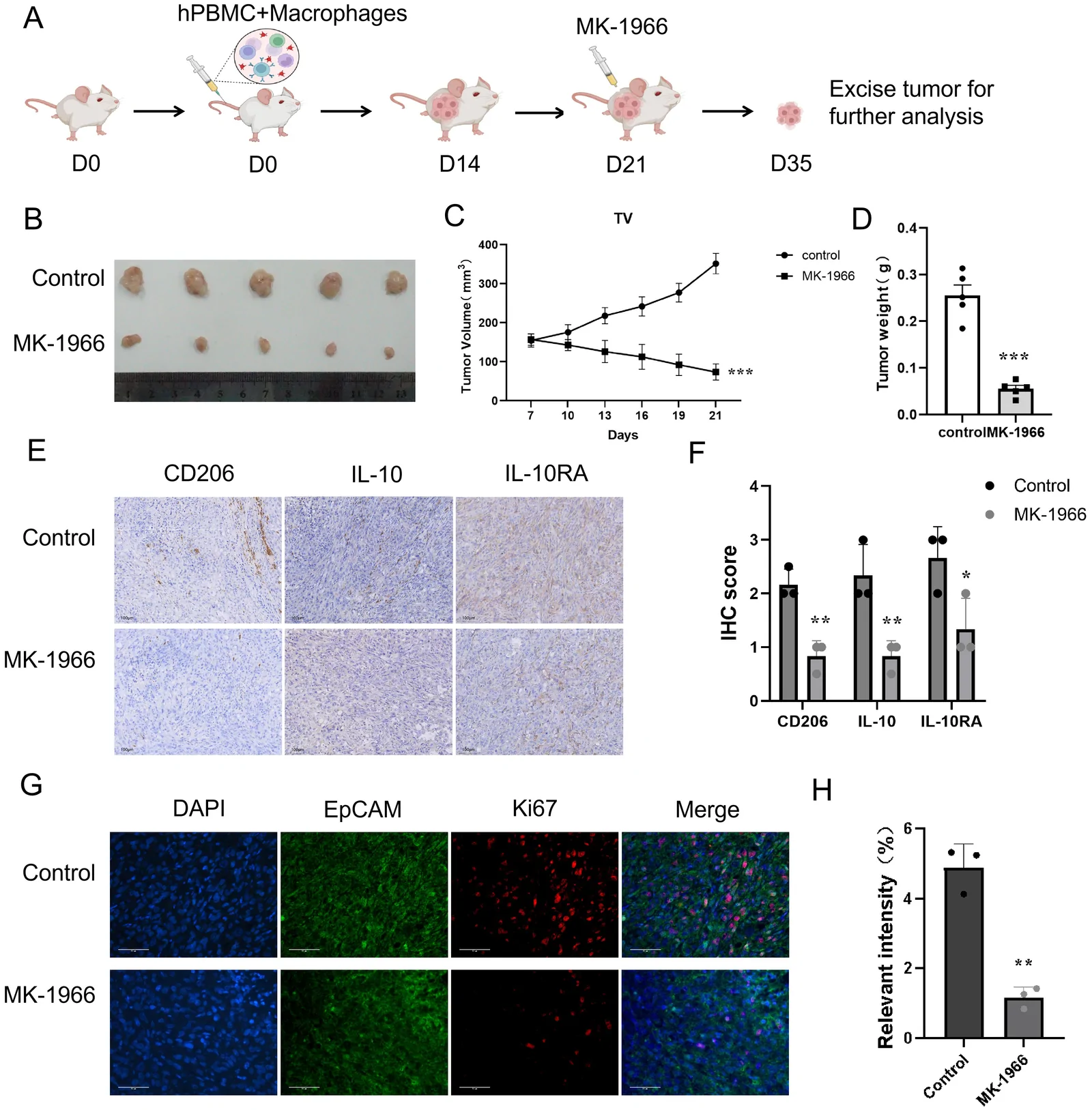
IL10 blockade with MK-1966 suppresses tumor growth and decreases IL10 pathway activity in humanized PDX models. **(A)** Schematic of humanized ovarian cancer PDX model generation and MK-1966 treatment timeline. **(B)** Representative tumor images demonstrating visibly smaller tumors in MK-1966–treated mice. **(C)** Tumor growth curves showing significantly reduced tumor volume following MK-1966 treatment (n = 5, *p* < 0.001). **(D)** Final tumor weight demonstrating significantly lower tumor mass in treated mice (n = 5, *p* < 0.001). **(E)** Representative IHC staining showing decreased CD206, IL-10, and IL-10RA expression in MK-1966 tumors versus controls. **(F)** Quantitative IHC scoring confirming significant reductionsin CD206 (p<0.01) and IL-10 and IL-10RA (*p* < 0.05) following treatment. N = 3 **(G)** Representative multiplex immunofluorescence staining of DAPI (blue), EpCAM (green), and Ki67 (red) demonstrating co-localization of Ki67-positive proliferating cells within EpCAM-positive tumor cells. MK-1966 treatment markedly reduced EpCAM^+^/Ki67^+^ double-positive tumor cells compared with controls. **(H)** Quantitative analysis of EpCAM^+^/Ki67^+^ relative fluorescence intensity demonstrating significantly decreased tumor-cell proliferative activity following MK-1966 treatment (**p< 0.01). * represent p-value < 0.05; *** represent p-value < 0.001.

The treatment schema is shown in **Figure 6A**, illustrating the generation of humanized mice using human PBMCs followed by establishment of ovarian cancer PDX tumors and subsequent MK-1966 administration. In immune-humanized ovarian cancer PDX mice,treatment with the IL10 monoclonal antibody MK-1966 resulted invisibly smaller tumors compared with controls (**Figure 6B**). Longitudinal tumor monitoring demonstrated a progressive reduction in tumor volume in the MK-1966 group, whereas tumors in control mice continued to enlarge, with a significant difference observed by day 21 (*p*< 0.001, **Figure 6C**). Final tumor weight measurements confirmed this effect, showing substantially lower tumor mass in MK-1966–treated mice compared with controls (*p* < 0.001, **Figure 6D**). Immunohistochemical analysis revealed marked reductions in CD206, IL10, and IL10RA staining in tumors from MK-1966–treated mice relative to controls (**Figure 6E**). Quantitative scoring demonstrated significantly decreased expression of CD206 (p< 0.01), and reduced IL10 and IL10RA expression (p< 0.05) in treated tumors (**Figure 6F**). These results showed that MK-1966 treatment is associated with reduced tumor burden and decreased expression of IL10 pathway components and M2-associated markers in humanized ovarian cancer PDX tumors. To further determine whether MK-1966 reduced proliferation specifically in tumor cells, multiplex immunofluorescence costaining was performed using the epithelial tumor marker EpCAM together with Ki67. In control tumors, abundant Ki67-positive nuclei colocalized with EpCAM-positive tumor cells, indicating active proliferation within the malignant epithelial compartment (**Figure 6G**). In contra st, MK-1966 treatment markedly reduced the proportion of EpCAM+/Ki67+ double-positive cells. Quantitative analysis.demonstrated a significant decrease in the relative intensity of proliferating EpCAM-positive tumor cells in the MK-1966 group compared with controls (*p* < 0.01, **Figure 6H**). These findings further support that IL10 blockade suppresses ovarian tumor progression by reducing tumor-cell proliferative activity and attenuating immunosuppressive IL10-associated signaling within the tumor microenvironment.

## Discussion

Our study delineates a previously uncharacterized fibroblast–macrophage immunoregulatory axis in HGSOC-TME, highlighting the dominant role of IL-10 signalling in shaping immune suppression and tumor progression. Using scRNA-seq coupled with cell–cell communication inference, we identified a transcriptionally distinct population of iCAFs that produce IL-10 and engage macrophages enriched for IL-10 receptor expression, reinforcing an immunosuppressive milieu within ascites. This axis aligns with emerging evidence that IL-10 and immunosuppressive macrophages jointly contribute to tumor immune evasion and poor clinical outcomes in ovarian cancer. Multiple studies have established that ovarian cancer ascites harbors an immunosuppressive cytokine milieu, with IL-10 consistently present at high levels relative to serum and correlated with disease progression and reduced survival (19, 20). Ascitic IL-10 has been shown to impair antigen presentation, suppress effector T-cell activation, and promote regulatory T-cell activity, contributing to immune evasion (21). These observations are consistent with our finding that IL-10 is predominantly produced by iCAFs within HGSOC ascites, rather than by tumor epithelial cells or lymphoid populations, indicating that stromal elements have substantial immunomodulatory influence. Tumor-associated macrophages are among the most abundant immune cell populations in ovarian cancer and are known to exhibit functional plasticity, with M2-like TAMs linked to poor prognosis, chemoresistance, and metastatic progression (22, 23). M2-like macrophages produce anti-inflammatory mediators such as IL-10 and TGF-β, which suppress cytotoxic immune responses and support tumor survival through diverse pro-tumorigenic mechanisms including angiogenesis, extracellular matrix remodelling, and extracellular vesicle signalling (23, 24). Our analysis revealed that IL-10 receptor expression (IL-10RA and IL-10RB) is significantly enriched in M2-macrophages compared with M1 macrophages, indicating a heightened capacity for IL-10 responsiveness in the immunosuppressive macrophage subset. This observation is consistent with prior reports indicating that M2 TAMs preferentially respond to immunosuppressive signals within the TME, contributing to an environment that favors tumor growth and immune escape.

The spatial co-localization of IL-10–producing iCAFs with IL-10R–expressing M2 macrophages in tumor tissues supports a model of localized intercellular communication. Such spatial proximity likely facilitates efficient cytokine signaling, amplifying immunosuppressive effects at the tumor–stroma interface. This spatial organization mirrors findings in other solid tumors, where tight coupling between stromal fibroblasts and macrophages fosters immune tolerance and disease progression. For example, functional spatial analyses have demonstrated that cancer-associated fibroblast (CAF) clusters are enriched in immunosuppressive compartments that recruit or polarize TAMs toward a pro-tumoral phenotype (25–27). The correlation of the IL-10R-high M2 macrophage signature with immune exhaustion markers further underscores the functional relevance of this axis. Specifically, the strong association between our IL-10R-high M2 signature and exhausted T-cell infiltration suggests that IL-10–driven macrophage programs may contribute to T-cell dysfunction in ovarian cancer. This aligns with previous work showing that immunosuppressive TAM phenotypes correlate with exhausted cytotoxic T-cell states across multiple cancer types. High M2/M1 macrophage ratios have been associated with reduced survival in ovarian cancer, likely through suppression of effector immune cytotoxicity and promotion of T-cell exhaustion (22, 28, 29). The weaker correlation between the IL-10R-high signature and total CD8^+^ T-cell infiltration may reflect the functional state of these T-cells; it is not the absolute number but the exhaustion phenotype that is more tightly linked to immunosuppression, as has been observed in multiple human cancers. M2-like macrophages in tumours often share transcriptional programs with wound-healing macrophages, reflecting tissue-remodelling and immunoregulatory functions that may be co-opted by tumours to support progression.

The paracrine signalling between IL-10–producing fibroblasts and IL-10R–expressing macrophages may also have implications for key intracellular signaling pathways within TAMs themselves. IL-10 is known to signal via the JAK1/STAT3 axis following dimerization with its receptors, leading to transcriptional programs that favor immune regulation and anti-inflammatory states (30). STAT3 activation in TAMs has been implicated in promoting pro-tumoral functions including suppression of antigen presentation and expression of co-inhibitory molecules, which may underlie the enrichment of exhausted immune states associated with the IL-10R-high signature. Our findings also align with preclinical evidence showing that neutralization of IL-10 can reverse immunosuppressive programs. In gastric cancer models, IL-10 neutralizing antibodies partially inhibited tumor-promoting effects of macrophage-conditioned media, suggesting that IL-10 blockade may impair tumor–stroma communication and diminish M2 polarization (31, 32). In our humanized ovarian cancer PDX model, IL-10 blockade with MK-1966 led to significant inhibition of tumor growth and reduced expression of macrophage-associated and IL-10 pathway markers, including CD206, IL10, and IL10RA. Importantly, because Ki67 is not exclusively restricted to malignant cells, we further performed multiplex immunofluorescence co-localization analysis using the epithelial tumor marker EpCAM together with Ki67. This analysis demonstrated that proliferative Ki67 signals were predominantly localized within EpCAM-positive tumor cells and were markedly reduced following MK-1966 treatment, confirming that IL-10 blockade suppresses proliferation specifically within the malignant epithelial compartment. These findings strengthen the interpretation that disruption of IL-10 signaling not only attenuates immunosuppressive macrophage polarization but also directly associates with reduced tumor-cell proliferative activity within the ovarian cancer microenvironment. Importantly, the interplay between iCAFs and TAMs adds to a growing body of literature recognizing cancer-associated fibroblasts as active modulators of immune responses rather than passive structural supporters. CAFs secrete a range of cytokines and extracellular matrix molecules that critically regulate immune cell recruitment, differentiation, and localization within tumors. Notably, fibroblast-derived CXCL12 has been shown to mediate T-cell exclusion and immune evasion in pancreatic cancer, where pharmacologic blockade of the CXCL12–CXCR4 axis restored T-cell infiltration and enhanced immunotherapy response (33). Our identification of IL-10 as a fibroblast-derived mediator positions iCAFs as key orchestrators of immune suppression via direct macrophage modulation. Similar CAF-driven chemokine networks have been implicated in ovarian cancer, where stromal chemokine signalling contributes to immune suppression (34).

The clinical relevance of IL-10 signalling in ovarian cancer is further supported by studies showing that elevated IL-10 levels in ascitic fluid and serum are associated with advanced tumor stage and poorer clinical outcomes, with higher IL-10 concentrations correlating with shortened progression-free survival and reduced overall survival in patients with epithelial ovarian carcinoma (14, 15). These clinical associations, combined with our mechanistic data, underscore the potential importance of IL-10 not only as a biomarker of immunosuppressive TME states but also as a therapeutic target. Our data contribute to a refined understanding of the cellular communication networks that shape the immunosuppressive landscape of HGSOC ascites, highlighting a specific stromal–myeloid axis driven by IL-10 that reinforces macrophage polarization and immune exhaustion. These insights underscore the complex crosstalk between non-malignant stromal elements and innate immune cells in promoting tumor progression and may inform future strategies aimed at remodelling the TME to enhance anti-tumor immunity.

This study provides a robust multi-modal demonstration of a stromal–myeloid IL-10–IL-10RA axis in HGSOC; however, several aspects warrant further refinement. Although we utilized publicly available scRNA-seq data combined with spatial validation and functional immune-humanized PDX modelling, the cohort size remains modest and may not capture the full breadth of inter-patient heterogeneity. In addition, the sample size used for the *in vivo* validation experiments was relatively small, and the immunohistochemical staining analysis provides supportive but limited evidence of the IL-10 pathway modulation in tumor tissues. Larger prospective cohorts and multi-centre validation studies would therefore strengthen the generalizability of these findings. Mechanistically, while our data strongly support iCAF-derived IL-10 signalling toward IL-10RA-high M2 macrophages, direct lineage-tracking experiments and *in vitro* macrophage–fibroblast coculture systems would further clarify causal directionality and temporal dynamics. Future studies incorporating flow cytometry–based immune profiling and coculture models will be important to more precisely evaluate the IL-10–IL-10R signaling axis and its role in macrophage polarization. Additionally, the immune-humanized PDX model approximates but does not fully replicate human immunity, and future evaluation in more advanced translational models and ultimately early-phase clinical studies will be critical to assess therapeutic applicability. Nonetheless, these limitations do not diminish the strength of the biological signal identified; instead, they highlight promising avenues for therapeutic exploration, rational combination immunotherapy design, and biomarker development.

## Conclusion

This study delineates a dominant IL-10–IL-10RA signalling axis linking IL-10-producing iCAFs with IL-10RA/IL-10RB-enriched M2-macrophages, defining a key stromal–myeloid communication pathway that shapes the immunosuppressive architecture of the HGSOC tumor microenvironment. The IL-10R-high macrophage transcriptional program associates strongly with immune exhaustion and macrophage enrichment, and this cellular interaction is reinforced by spatial colocalization in tumor tissues. Functional blockade of IL-10 using MK-1966 significantly suppressed tumor growth and reduced IL-10 pathway activity and M2-associated markers in immune-humanized PDX models, supporting the therapeutic relevance of targeting this pathway. Collectively, these findings identify IL-10 signalling as a central stromal-immune axis in HGSOC and provide a compelling rationale for IL-10–directed therapeutic strategies to remodel the tumor microenvironment and enhance anti-tumor immunity.

## Data Availability

The datasets presented in this study can be found in online repositories. The names of the repository/repositories and accession number(s) can be found in the article/Supplementary Material.
