# Correction: Single-cell and spatial profiling reveal an IL-10–associated iCAF–M2 macrophage communication axis in high-grade serous ovarian cancer ascites

**DOI:** 10.3389/fimmu.2026.1946632

**Published:** 2026-09-18

**Authors:** Ren Li, Jinquan Xia, Chang Zou, Yixia Xie, Yuan Shen

**Affiliations:** 1 The Second Clinical Medical College, Jinan University (Shenzhen People’s Hospital), Shenzhen, Guangdong, China; 2 Department of Clinical Research Centre, Shenzhen People’s Hospital (The First Affiliated Hospital, Southern University of Science and Technology; The Second Clinical Medical College, Jinan University), Shenzhen, Guangdong, China; 3 Department of Intensive Care Unit, Shenzhen People’s Hospital (The First Affiliated Hospital, Southern University of Science and Technology; The Second Clinical Medical College, Jinan University), Shenzhen, Guangdong, China; 4 Department of Gynecology, The First Affiliated Hospital of Jinan University, Guangzhou, Guangdong, China

**Keywords:** fibroblast–macrophage crosstalk, high-grade serous ovarian cancer, IL-10–IL-10RA signalling axis, immune-humanized PDX therapy, single-cell transcriptomics

There was a mistake in **Figure 6** as published. “Panel H” was not fully uploaded during the proof stage. The corrected **Figure 6** appears below.

**Figure 6:**
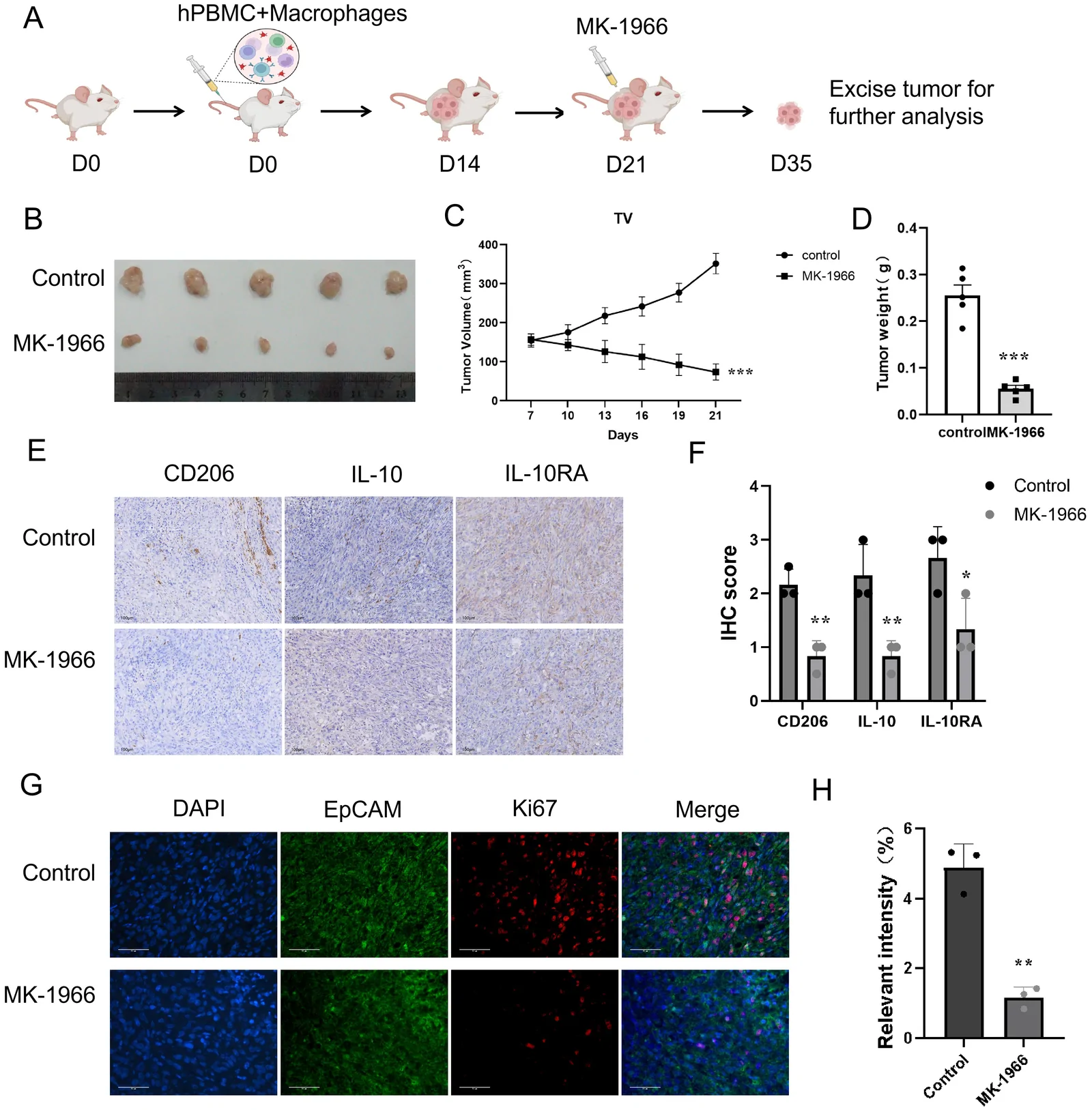
IL10 blockade with MK-1966 suppresses tumor growth and decreases IL10 pathway activity in humanized PDX models. **(A)** Schematic of humanized ovarian cancer PDX model generation and MK-1966 treatment timeline. **(B)** Representative tumor images demonstrating visibly smaller tumors in MK-1966–treated mice. **(C)** Tumor growth curves showing significantly reduced tumor volume following MK-1966 treatment (n = 5, p < 0.001). **(D)** Final tumor weight demonstrating significantly lower tumor mass in treated mice (n = 5, p < 0.001). **(E)** Representative IHC staining showing decreased CD206, IL-10, and IL-10RA expression in MK-1966 tumors versus controls. **(F)** Quantitative IHC scoring confirming significant reductions in CD206 (p < 0.01) and IL-10 and IL-10RA (p < 0.05) following treatment. N = 3. **(G)** Representative multiplex immunofluorescence staining of DAPI (blue), EpCAM (green), and Ki67 (red) demonstrating co-localization of Ki67-positive proliferating cells within EpCAM-positive tumor cells. MK-1966 treatment markedly reduced EpCAM+/Ki67+ double-positive tumor cells compared with controls. **(H)** Quantitative analysis of EpCAM+/Ki67+ relative fluorescence intensity demonstrating significantly decreased tumor-cell proliferative activity following MK-1966 treatment (**p< 0.01). *represent p-value < 0.05; *** represent p-value < 0.001.

The original version of this article has been updated.

There was a mistake in the caption of **Figure 6** as published. We conducted multiplex immunofluorescence staining on tumor tissues from a mouse PDX model. By detecting Ki67 and EpCAM in tumor tissues, we aimed to elucidate the impact of blocking the IL-10 signaling axis on tumor tissues. Therefore, the original description of immunohistochemical scoring for Ki67 was no longer necessary. The corrected caption of **Figure 6** appears below.

“(E) Representative IHC staining showing decreased CD206, IL-10, and IL-10RA expression in MK-1966 tumors versus controls.

(F) Quantitative IHC scoring confirming significant reductions in CD206 (p < 0.01) and IL-10 and IL-10RA (p < 0.05) following treatment. N = 3.”

The original version of this article has been updated.

“We conducted multiplex immunofluorescence staining on tumor tissues from a mouse PDX model. By detecting Ki67 and EpCAM in tumor tissues, we aimed to elucidate the impact of blocking the IL-10 signaling axis on tumor tissues. Therefore, the original description of immunohistochemical scoring for Ki67 was no longer necessary.”

A correction has been made to the section **Results**, *IL10 blockade with MK-1966 suppresses tumor growth in humanized PDX models*, Paragraph 1:

“The treatment schema is shown in **Figure 6A**, illustrating the generation of humanized mice using human PBMCs followed by establishment of ovarian cancer PDX tumors and subsequent MK-1966 administration. In immune-humanized ovarian cancer PDX mice,treatment with the IL10 monoclonal antibody MK-1966 resulted invisibly smaller tumors compared with controls (**Figure 6B**). Longitudinal tumor monitoring demonstrated a progressive reduction in tumor volume in the MK-1966 group, whereas tumors in control mice continued to enlarge, with a significant difference observed by day 21 (p< 0.001, **Figure 6C**). Final tumor weight measurements confirmed this effect, showing substantially lower tumor mass in MK-1966–treated mice compared with controls (p < 0.001, **Figure 6D**).Immunohistochemical analysis revealed marked reductions in CD206, IL10, and IL10RA staining in tumors from MK-1966–treated mice relative to controls (**Figure 6E**). Quantitative scoring demonstrated significantly decreased expression of CD206 (p< 0.01), and reduced IL10 and IL10RA expression (p< 0.05) in treated tumors (**Figure 6F**). These results showed that MK-1966 treatment is associated with reduced tumor burden and decreased expression of IL10 pathway components and M2-associated markers in humanized ovarian cancer PDX tumors. To further determine whether MK-1966 reduced proliferation specifically in tumor cells, multiplex immunofluorescence costaining was performed using the epithelial tumor marker EpCAM together with Ki67. In control tumors, abundant Ki67-positive nuclei colocalized with EpCAM-positive tumor cells, indicating active proliferation within the malignant epithelial compartment (**Figure 6G**). In contra st, MK-1966 treatment markedly reduced the proportion of EpCAM+/Ki67+ double-positive cells. Quantitative analysis. demonstrated a significant decrease in the relative intensity of proliferating EpCAM-positive tumor cells in the MK-1966 group compared with controls (p < 0.01, **Figure 6H**). These findings further support that IL10 blockade suppresses ovarian tumor progression by reducing tumor-cell proliferative activity and attenuating immunosuppressive IL10-associated signaling within the tumor microenvironment.”

The original version of this article has been updated.

